# O.4.4-10 Policy and environmental correlates of physical activity in Croatia

**DOI:** 10.1093/eurpub/ckad133.207

**Published:** 2023-09-11

**Authors:** Anja Maria Jukić, Fran Žganec Brajša, Danijel Jurakić

**Affiliations:** Faculty of Kinesiology, Croatia; anja.m.jukic.18.6@gmail.com; Faculty of Kinesiology, Croatia; anja.m.jukic.18.6@gmail.com; Faculty of Kinesiology, Croatia; anja.m.jukic.18.6@gmail.com

## Abstract

**Purpose:**

Although there are several studies that have determined the correlates of physical activity in the Republic of Croatia, policy and environmental correlates are still unknown. Therefore, we conducted a study with the main goal to examine associations of sport and exercise participation with perception of policy and environmental factors in Croatia, including opportunities for engaging in physical activities, the sports clubs offer, and level of the support from local authorities.

**Methods:**

The study was conducted on 1031 randomly selected Croatian citizens using questionnaire containing questions about sport and exercise, participation, engagement opportunities for physical activities, the sports clubs’ and other service providers’ offer and level of the support from local authorities. In the multivariate logistic regression, participation in sports and exercise was the dependent variable, and the independent variables were perception of opportunities for physical activity, perception of local sports clubs’ and other service providers’ offer, and perception of local government support for physical activity.

**Results:**

According to the findings of the logistic regression analysis, local sports clubs’ and other service providers’ offer as well as government support were identified as significant predictors of engagement in sport and exercise. To be specific, individuals who perceive their local sports clubs and other service providers as providing a greater range of opportunities, as well as those who feel that their local government supports physical activity, are more inclined to engage in sports and exercise activities. (p < 0.05).

**Conclusion:**

Future strategies aimed to motivate citizens to engage in sports and exercise should be based on encouraging local authorities to collaborate with sports clubs and other service providers to create an enabling environment that supports physical activity participation. This could include providing funding and resources to sports clubs and service providers to enable them to offer a wider range of opportunities that cater to the diverse interests and needs of individuals in the community.

**Funding:**

This work was supported by the Erasmus the “Creating Mechanisms for Continuous Implementation of the Sports Club for Health Guidelines in the European Union” (SCforH 2020-22) project, funded by the Erasmus+ Collaborative Partnerships grant (ref: 613434-EPP-1-2019-1-HR-8 SPO-SCP).

